# Primary Splenic Diffuse Large B-Cell Lymphoma: A Rare Case of Massive Splenomegaly and Thrombocytopenia

**DOI:** 10.7759/cureus.3026

**Published:** 2018-07-22

**Authors:** Sidra Khalid, Jad H Daw, Hamed Daw, Abdo Haddad

**Affiliations:** 1 Internal Medicine/Residency, Fairview Hospital/Cleveland Clinic, Cleveland, USA; 2 School of Arts and Sciences, Case Western Reserve University, Cleveland, USA; 3 Hematology and Oncology, Fairview Hospital/Cleveland Clinic, Cleveland, USA; 4 Department of Hematology and Oncology, Fairview Hospital/Cleveland Clinic, Cleveland, USA

**Keywords:** primary splenic dlbcl, splenomegaly, thrombocytopenia, r-chop, splenectomy, hcv

## Abstract

Primary splenic diffuse large B-cell lymphoma (DLBCL) is a rare type of non-Hodgkin’s lymphoma. It often presents with abdominal pain or splenomegaly. We present a case of a 68-year-old male who presented to the emergency department with left sided abdominal pain. Workup revealed massive splenomegaly and thrombocytopenia. A splenic biopsy confirmed the diagnosis of primary splenic DLBCL. The patient was treated with chemotherapy. This case highlights the importance of considering primary splenic DLBCL with splenomegaly and treating it with chemotherapy and/or splenectomy.

## Introduction

Primary splenic diffuse large B-cell lymphoma (DLBCL) is a rare type of non-Hodgkin’s lymphoma. It commonly presents with abdominal pain, splenic mass and an elevated lactate dehydrogenase level [1]. Diagnosis is based on imaging findings of splenomegaly with hypoenhancing masses on ultrasound or CT (computed tomography) scan. Treatment options include splenectomy, chemotherapy and/or splenic irradiation [2]. Hepatitis C virus (HCV) is associated with early-stage DLBCL and should be checked in patients [3]. In this case, we will describe the diagnostic approach to a patient with splenomegaly, the clinical course of a patient with primary splenic DLBCL, and the treatment options we decided to pursue based on the patient’s co-morbid conditions, performance status, and stage of the disease.

## Case presentation

**Figure 1:**
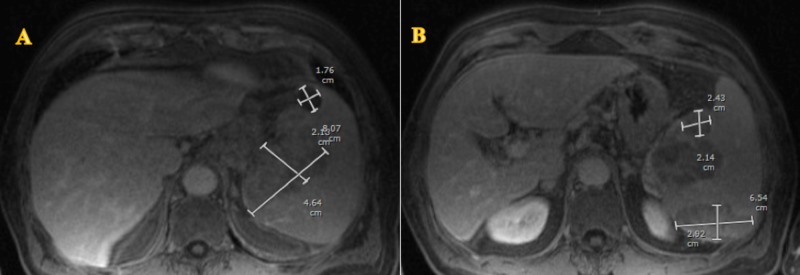
(A & B) Magnetic resonance imaging (MRI) of the abdomen showing splenomegaly with multiple hypoenhanced lobulated masses.

A 68-year-old male presented to the emergency department with left-sided abdominal pain, early satiety, fatigue and 7 lbs weight loss for seven weeks. His past medical history was significant for hypertension, emphysema and HCV with an undetectable HCV ribonucleic acid (RNA) following treatment with sofosbuvir and ribavirin. Vital signs were stable and clinical examination was unremarkable, except for left-sided abdominal tenderness. His labs showed a hemoglobin of 16.4 mg/dL, white blood cell count of 6.31 k/uL, and platelet count of 101 k/uL with normal liver function tests. Abdominal ultrasound showed splenomegaly and three heterogeneous hypoechoic masses in the spleen. Magnetic resonance imaging (MRI) of the abdomen confirmed splenomegaly with multiple hypoenhanced lobulated masses in the spleen; no evidence of lymphadenopathy or hepatomegaly (Figure 1A, 1B). Single-photon emission computerized tomography (SPECT) identified heterogeneous splenic uptake with areas of photopenia related to splenic masses.

Since the patient had a history of HCV, we needed to rule out liver disease leading to splenomegaly. His liver biopsy showed chronic hepatitis of mild activity with focal bridging fibrosis. Bone marrow biopsy revealed no evidence of lymphoma. Janus Kinase 2 (JAK 2) mutation was negative. Hence, liver disease was not the main cause, and thrombocytopenia was attributed to hypersplenism. Therefore, splenic biopsy was performed by interventional radiology, which showed DLBCL. The patient was started on rituximab, cyclophosphamide, doxorubicin, vincristine, and prednisone (R-CHOP). The decision was made to delay splenectomy due to massive splenomegaly and a possible bleed inside the spleen as the patient was recently started on warfarin for new onset atrial fibrillation. After six cycles of R-CHOP, repeated imaging with CT abdomen and positron emission tomography (PET) scan showed complete remission on PET and decrease in spleen size. Subsequently, after six months, he presented with left lower quadrant pain and splenic biopsy showed a relapse of DLBCL. Due to his poor performance status, he was not a candidate for autologous stem cell transplant and was started on palliative lenalidomide and rituximab.

## Discussion

Primary splenic DLBCL is very rare as it occurs in less than 1% of non-Hodgkin’s lymphomas [2]. It is most commonly found in females and older males [4]. Symptoms can include splenomegaly as well as left upper quadrant pain, fever and weight loss. It can also be associated with human immunodeficiency virus (HIV), and can present with metastasis to hilar and retroperitoneal lymph nodes [5]. It also causes cytopenias and elevated levels of erythrocyte sedimentation rate and B2 microglobulin. Primary splenic DLBCL is classified in three stages. Stage I refers to when it is confined to the spleen, stage II refers to the involvement of the spleen and hilar lymph nodes, and stage III refers to extra-splenic nodal or hepatic involvement [2]. Upon imaging studies, primary splenic DLBCL appears as hypodense splenic regions on contrast-enhanced CT scans, or hypoechoic lesions on ultrasound. Splenic biopsy or splenectomy is required for definite diagnosis. Options for treatment include splenectomy only, splenectomy and chemotherapy with R-CHOP, or radiation therapy with chemotherapy [2].

Bairey et al. studied 87 patients with primary splenic DLBCL and concluded that patients who had a low eastern cooperative oncology group performance status and splenectomy had a better progression-free survival. When splenectomy was performed in early-disease patients at diagnosis, it resulted in better survival outcomes [1]. Brox et al. studied nine patients with primary splenic lymphoma and saw a median survival time of 7.48 years who underwent splenectomy alone or a splenectomy with chemotherapy. They found no correlation between histological subtype and prognosis [6]. Splenic irradiation is also a treatment option for inoperable cases in order to reduce the size of the spleen [7,8].

Additionally, Iannitto and Tripodo had suggested that treatment for DLBCLs involving the spleen should be guided by the recommendations proposed for other types of DLBCLs. For example, in patients who undergo splenectomy and are diagnosed with primary splenic DLBCL, they should be treated as DLBCL patients with surgically resected extranodal disease. They would receive four cycles of R-CHOP. Subsequently, restaging would be performed in addition to two more cycles of R-CHOP to achieve complete remission [9].

Relapse is a common problem with those who suffer from DLBCL, as 40% of those with DLBCL will eventually relapse [4]. Relapse could be diagnosed with whole body F-deoxyglucose (FDG) PET scan or a splenic biopsy [7]. Mondello et al. conducted a retrospective study on 123 patients with relapsed or refractory DLBCL, which showed that lenalidomide at 25 mg/day is a superior therapeutic option than 15 mg/day and it is more effective in patients with non-germinal center B-cell DLBCL [10].

Moreover, Yu and Lin reviewed clinicopathology of 74 spleens with splenic lymphoma and HCV serology. They found that HCV was associated with early stage DLBCL. The role of HCV infection in causing lymphoma is hypothesized through several mechanisms. First, the infection causes chronic antigen stimulation causing lymphocytic proliferation. Second, the HCV itself may infect lymphocytes directly. Third, the HCV-encoded E2 protein may cause CD81 to activate B-cells, reduce apoptosis, and increase double DNA strand breaks. Hence, further studies are needed to determine the pathogenesis of HCV in primary splenic DLBCL [3].

## Conclusions

In cases of massive splenomegaly, primary splenic DLBCL should be considered in the differential diagnosis. Effective treatment options include splenectomy, chemotherapy and/or radiation. For patients who have HCV and present with splenomegaly, it is important to consider DLBCL as HCV is associated with it.
